# University students’ fertility awareness and its influencing factors: a systematic review

**DOI:** 10.1186/s12978-023-01628-6

**Published:** 2023-06-06

**Authors:** Yue Ren, Yue Xie, Qulian Xu, Miaochen Long, Ying Zheng, Lin Li, Changmin Niu

**Affiliations:** 1 School of Nursing School of Public, Health Yangzhou University, Mid Jiangyang Road 136, Yangzhou, Jiangsu People’s Republic of China; 2 Department of Obstetrics and Gynecology, Yangzhou University Affiliated Hospital, Yangzhou, China

**Keywords:** Fertility awareness, Influencing factors, College students, Age-related fertility decline, Systematic review

## Abstract

**Introduction:**

In recent years, a growing number of researchers have begun to study fertility awareness (FA). Evidence suggests that college students in their reproductive years have a common understanding of fertility, risk factors for infertility, and assisted reproductive technologies. Therefore, this systematic review summarizes these studies and explores the factors affecting college students’ fertility awareness.

**Methods:**

A systematic literature search of databases (PUBMED/MEDLINE, Cochrane, Web of Science, Embase, and EBSCO) was conducted from inception to September 2022. Studies that assessed the levels of fertility awareness and factors influencing college students were considered for the review. The qualities of the included studies were evaluated using the Strengthening the Reporting of Observational Studies in Epidemiology guidelines. This systematic review is reported according to the preferred reporting items for systematic review (PRISMA) guidelines.

**Results:**

Twenty-one articles met the eligibility criteria and were included. The preliminary results showed that participants reported low to moderate FA. Female medical students demonstrated higher levels of fertility awareness. The association between age, years of education, and FA was insufficient.

**Conclusion:**

The results of the current study suggest that increased FA interventions are warranted, especially for the male, non-medical student population. Governments and educational institutions should strengthen education programs for young students on reproductive health to help them raise awareness about childbirth, and society should provide family support for young people.

**Supplementary Information:**

The online version contains supplementary material available at 10.1186/s12978-023-01628-6.

## Introduction

World Health Organization (WHO) defines infertility as the inability to conceive after at least 12 months of regular sexual life without contraception. About 12–15% of couples worldwide suffer from infertility, a significant health problem worldwide. Globally, the time to the first conception is increasing for both men and women, while overall fertility is declining. Some studies have shown that people delay childbearing for reasons such as pursuing a career, pursuing further education, financial reasons, and finding a suitable partner [1]. However, human fertility declines with age, and there are many risk factors; if young people of childbearing age are unaware of the problem, they are likely to experience involuntary childlessness [2].

Insufficient fertility knowledge is a factor in the failure to achieve parenting goals [3], and fertility knowledge influences the decisions related to reproductive health. Many modifiable health risk factors can also lead to infertility. Declining fertility, delayed fertility, and increased infertility have led researchers worldwide to assess fertility awareness (FA). This concept was defined in the International Glossary of Infertility and Fertility Care in 2017 as "the understanding of reproduction, fertility, associated individual risk factors (for example, advanced age, sexual health factors, and sexually transmitted infections), and lifestyle factors (such as smoking and obesity), as well as non-personal risk factors (for instance, environmental and workplace factors). It includes an awareness of the social and cultural factors influencing family planning choices and the social and cultural factors affecting the need for family building [4].

College students, as a particular group of young people, have been scrutinized because they are at their optimal reproductive age and face choices between education, career, marriage, and childbearing. Most studies on FA show that college students of childbearing age are eager to start a family and have children. However, they do not know much about the optimal age to have children, age-related fertility decline, and risk factors for infertility. The lack of FA has made many countries aware of the importance of reproductive health education. As a result, UK-based academics have launched an initiative to develop tools and information for teenagers, adults, teachers, parents, and health professionals to raise awareness of reproductive health [5–8].

However, no review has systematically evaluated the FA level among college students and its influencing factors. Therefore, in this systematic review, we elaborated on the level of FA among college students of childbearing age and identified the influencing factors.

## Methods

This systematic review complied with the Preferred Reporting Items for Systematic Reviews and Meta-Analyses (PRISMA) statement (Additional file 1). The protocol of this review was registered in the International Prospective Register of Systematic Reviews (registration number: CRD42022372075).

### Research strategy

A comprehensive literature search was conducted in the PubMed, Web of Science, Cochrane, Embase, and EBSCO databases from inception to November 2022 for studies reporting the level of college students’ fertility awareness and its influencing factors. The literature search was limited to English publications only. The search terms in PubMed were as follows: (“fertility” OR “fertile period” OR “delayed childbearing” OR “trying ‘to’ conceive” OR “assisted reprod*”) AND (“awareness” OR “knowledge” OR “perception” OR “health knowledge, attitudes, practice” OR “fertility awareness” OR “fertility knowledge”) AND (“university student” OR “college student” OR “undergraduate”) AND (“risk factors” OR “related factors” OR “relevant factors” OR “influencing factors)) OR “influence factors” OR “affecting factors”). We also identified eligible articles from the references.

### Inclusion and exclusion criteria

Studies were included when they met the following criteria: (1) research subjects were University students; (2) quantitative data on FA (for example, age-related fertility decline, fertility risk factors, infertility definition, and intended behavior in the event of infertility, knowledge about IVF treatments, and influencing factors of fertility awareness) were used; (3) FA-specific measures were used, or the problem of the evaluation was described in detail.

Non-English publications, reviews, abstracts, communications, case reports, and studies that could not be provided in complete and non-human studies were excluded.

### Study selection

All searched articles were first imported into NoteExpress software to remove duplicates automatically. Then two reviewers screened the titles and abstracts of the studies according to inclusion and exclusion criteria to exclude irrelevant articles. Articles that met the preliminary eligibility criteria were subjected to full-text screening by the same two reviewers. Any disagreements were resolved by consulting with a third reviewer.

### Data extraction

The data were extracted using an Excel sheet designed in advance by two reviewers, and a third reviewer resolved any disagreements. The following parameters were recorded: (1) author and year of publication, (2) country, (3) study type, population, and sample size, (4) measurement tools used, (5) mean age, (6) education level, and (7) influencing factors. All information is summarized in Table 1.

**Table 1:** Characteristics of included studies

Authors, year	Country	Type of study, population and sample size	Measure used	Mean age	Education	Influencing factors
Ludmila Jurkowski, 2021 [6]	Argentina	Cross-sectional, 680 students (83.2% females and 16.4% males)	The awareness of fertility issues (Lampic et al. [7], the translation into Spanish)	Mean age: 24.7 years (SD = 5.6)	University	N/A
Lampic, 2006 [7]	Sweden	Cross-sectional, 401 students (222 females and 179 males)	The awareness of fertility issues [7]	Mean age: 24 years (SD = 4.0)	University	Gender
Laura Bunting, 2008 [8]	UK	Cross-sectional, 149 students (110 female and 39 male)	The factors affecting fertility scale (FAFS)	Mean age: 24.01 years (SD = 7.81)	Postgraduate and undergraduate university students	N/A
C. Meissner, 2016 [9]	Germany	Cross-sectional, 1144 students (881 female and 263 male)	Study-specific questionnaire	Mean age: 24.5 years	University (bachelor, master, doctor)	Gender, Major
Kazem Nouri, 2014 [10]	Germany	Prospective case–control study, 340 students (170 female and 170 male)	Study-specific questionnaire	Mean age: 20.03 years (SD = 1.77)	University (170 medical and 170 non-medical students)	Gender, Major
Brennan D. Peterson, 2012 [11]	USA	Cross-sectional, 246 students (138 female and 108 male)	The Swedish Fertility Awareness Questionnaire [7]	Mean age: 20.4 years (SD = 2.3)	Undergraduate university students	The difference between genders was not statistically significant
Valentina Rovei, 2010 [12]	Italy	Cross-sectional, 958 students (607 female and 351 male)	Study-specific questionnaire	Male mean age: 22.9 years; female mean age: 22.1 years	University students	Major
Karla L. Bretherick, 2010 [13]	Canada	Cross-sectional study,360 female students	Study-specific questionnaire	18–24 years	Undergraduate students	N/A
Aira Virtala, 2011 [14]	Finland	Cross-sectional, 4906 students (3222 female and 1684 male)	Study-specific questionnaire	Male mean age: 24.7 years; female mean age: 23.9 years	University students	Gender, Age
Carla Conceição, 2017 [15]	Portugal	Randomised controlled trial, 173students (intervention group: 89; control group: 84)	Educational video; the Swedish Fertility Awareness Questionnaire [7]	Mean age: 20.18 years (SD = 4.93)	University students	N/A
Hyewon Shin, 2020 [16]	Korean	Cross-sectional, 166 students (51.2% females and 48.8% males)	The Korean version of the Fertility Awareness Questionnaire and Attitudes of Parenthood (FAAP)	Mean age: 22.3 years (SD = 1.7)	University	N/A
C. H. Y. Chan, 2015 [17]	China	Cross-sectional, 367 students (275 females and 92 males)	The Swedish Fertility Awareness Questionnaire [7]	Mean age: 23.2 years (SD = 3.5)	University	N/A
J. M. Place, 2022 [18]	Mexican	Cross-sectional, 371 students (228 females and 143 males)	The Swedish Fertility Awareness Questionnaire [7]	Mean age: 23. 3 years (SD = 5.12)	University	N/A
Samaher Alfaraj, 2019 [19]	Saudi Arabia	Cross-sectional study, 248 female students	The Swedish Fertility Awareness Questionnaire [7]	Mean age: 21.36 years (SD = 1.35)	Undergraduate	The difference between genders was not statistically significant
Nina Olsén Sørensen, 2016 [20]	Danish	Cross-sectional, 517students (438 females and 79 males)	The Swedish Fertility Awareness Questionnaire [7]	Male mean age: 25.6 years (SD = 4.4); female mean age: 24.2 years (SD = 5.1)	Undergraduate	The difference between genders was not statistically significant
Ewelina Chawłowska, 2020 [21]	Polish	Cross-sectional study,456 female students	Study-specific questionnaire	Mean age: 21.95 years (SD = 2.45)	University	Gender, Major, Education
Agneta Skoog Svanberg, 2006 [22]	Sweden	Cross-sectional, 257students (141females and 116 males)	Study-specific questionnaire	N/A	Postgraduate students	N/A
Isidora, 2017 [23]	Serbian	Cross-sectional, 418 students (271 females and 147 males)	The Swedish Fertility Awareness Questionnaire [7]	Male mean age: 22.4 years (SD = 1.2); female mean age: 22.5 years (SD = 1.4)	University	The difference between genders was not statistically significant
Olumide Abiodun, 2016 [24]	Nigeria	Cross-sectional, 389 students (231 females and 158 males)	The Swedish Fertility Awareness Questionnaire [7]	Mean age: 18.74 years (SD = 2.14)	University undergraduates	N/A
Lisa C. Hickman, 2018 [25]	USA	Cross-sectional study,589female students	Study-specific questionnaire	N/A	Graduate students and medical trainees	Education
Ilse Delbaere, 2021 [26]	Europe (Sweden, Belgium and Greece)	Cross-sectional, 656 students (406 female and 247 male)	The Swedish Fertility Awareness Questionnaire [7]	Sweden: 25; Belgium: 25; Greece: 23	Medical undergraduate students	Gender, Major

### Quality assessment

The quality of each eligible article was independently assessed by two reviewers using the Guidelines for Strengthening Epidemiological Observational Studies (University of Bern, 2009). The guidelines comprised 22 items to evaluate the quality of cross-sectional and case–control articles. Each item was scored one if the study met the guidelines’ criteria and 0 if the study described the thing inadequately; the overall maximum score was 22 points. Studies with an overall score of ≥ 17 were considered high quality, those with an overall score between 11 and 16 were rated as moderate quality, and those with a total score of 10 were regarded as low quality. A quality assessment of 21 included quantitative studies did not identify any low-quality studies. Of these, 17 were considered high quality, while five studies were rated moderate. The distribution of scores is listed in Table 2.

**Table 2:** Quality evaluation results of included studies

	Title and abstract (1)	Introduction (2)	Methods (9)	Results (5)	Discussion (4)	Other information (1)	Score	Quality of evidence
Ludmila Jurkowski, 2021 [6]	1	2	6	3	3	1	16	Moderate
Lampic, 2006 [7]	0	2	7	4	4	0	17	High
Laura Bunting, 2008 [8]	0	2	7	4	3	1	17	High
Meissner, 2016 [9]	1	2	7	5	4	1	18	High
Kazem Nouri, 2014 [10]	1	2	6	4	3	0	16	Moderate
Brennan D. Peterson, 2012 [11]	0	2	7	3	4	1	17	High
Valentina Rovei, 2010 [12]	0	2	6	4	3	0	15	Moderate
Karla L. Bretherick, 2010 [13]	1	2	8	5	3	0	19	High
Aira Virtala, 2011 [14]	0	2	7	4	3	1	17	High
Carla Conceição, 2017 [15]	1	2	8	4	4	1	20	High
Hyewon Shin, 2020 [16]	1	2	8	3	4	0	18	High
C. H. Y. Chan, 2015 [17]	1	2	7	4	4	1	19	High
J. M. Place, 2022 [18]	1	2	6	4	4	0	17	High
Samaher Alfaraj, 2019 [19]	1	2	8	3	3	0	16	Moderate
Nina Olsén Sørensen, 2016 [20]	1	2	7	4	2	0	16	Moderate
Ewelina Chawłowska, 2020 [21]	0	2	7	4	4	1	18	High
Agneta Skoog Svanberg, 2006 [22]	0	2	8	4	3	0	17	High
Isidora, 2017 [23]	1	1	7	4	4	1	18	High
Olumide Abiodun, 2016 [24]	1	2	9	4	4	0	19	High
Lisa C. Hickman, 2018 [25]	1	2	7	5	4	1	20	High
Ilse Delbaere, 2021 [26]	1	2	7	5	4	1	20	High

## Results

### Search results

**Fig. 1:**
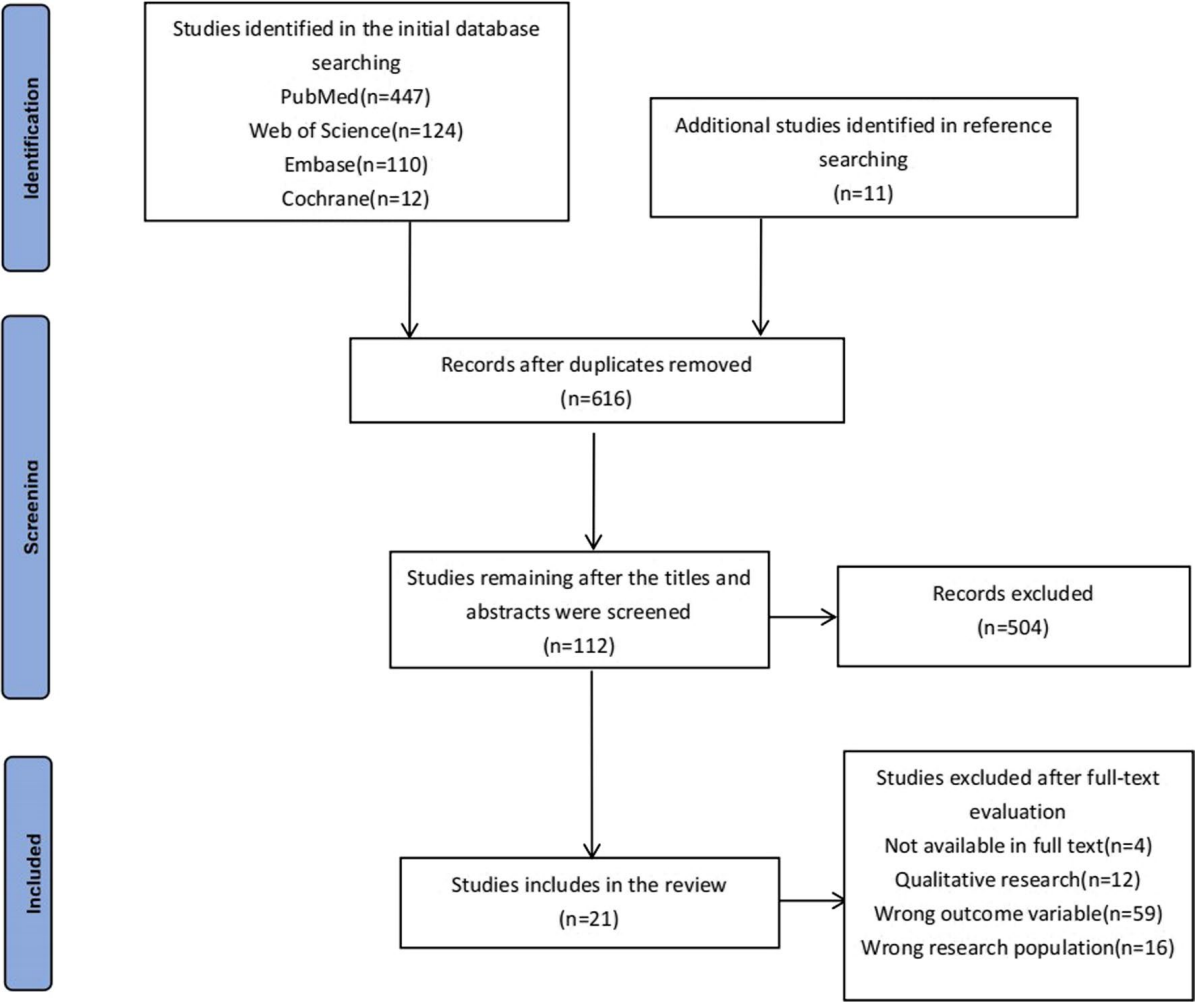
Prisma flowchart

Figure 1 summarizes the study selection process. A total of 704 studies were retrieved in the initial search, and 616 were retained after removing the duplicates. Subsequently, 504 studies were deemed irrelevant and excluded after screening the titles and abstracts. Next, we carefully reviewed the full text of 112 studies, of which 91 were excluded for various reasons. Finally, 21 studies were included in the systematic review.

### Study characteristics

Table 1 summarizes the features of the included studies published between 2006 and 2022. The sample sizes of the included studies ranged from 149 to 4906 individuals. All data were from 18 countries, with about 50% of the studies conducted in Europe (n = 12), 5 in the Americas, 3 in Asia, and 1 in Africa. Most were cross-sectional studies (n = 18), 2 were pre-test/post-test intervention studies, and 1 was a case–control study. The 21 studies included in the analysis focused on FA, but the measurement tools used were not identical, and the measurement methods were mostly self-reported questionnaires or interviews. Among them, the most commonly used is the FA scale developed by Lampic in 2006. The questionnaire mainly used different response scales and a format of true and false, multiple choice, or open-ended questions. None of the included studies reported global reproductive awareness scores. The topics explored included fertility risk factors, age-related fertility decline, infertility definition, intended behavior in the event of infertility, in vitro fertilization (IVF) treatment knowledge, and factors influencing FA.

### Overall FA

A total of 10 studies explicitly reported the level of FA among college students. In comparison, seven studies reported a marked lack of awareness of fertility issues among college students and low levels of FA [11, 12, 14, 16–18, 20, 25], and two studies reported high levels of FA [10, 26]. The remaining studies reported college students’ knowledge of human fertility and their knowledge of assisted reproductive technologies (ARTs).

### Specific dimensions of FA

#### Age-related fertility decline

A total of 16 studies analyzed the level of awareness of fertility declining with age. Among these, 14 reported low FA, indicating that college students overestimated the chances of having children at an older age [6, 7, 10–14, 16–19, 22–24], and only 2 had high FA, indicating that college students had a realistic view of fertility [25, 26].

Typically, there is a general lack of age-related awareness among students about declining human fertility. For example, research in Argentina showed that 36.2% of female students believe that female fertility declines between the ages of 45 and 50 years, 33.2% between the ages of 40 and 45 years, and 25.9% between the ages of 35 and 40 years; 57% of male students believed that male fertility does not decline with age [6]. A study in China showed that students overestimated the age of optimal fertility (69% of men, 79% of women), and they greatly overestimated the age at which female fertility began to decline (91% of men, 93% of women) [17]. Only a few participants realized that a slight decline in female fertility began before 30, and a significant decrease occurred in the late 30 s [7]. The two studies that reported high fertility awareness were conducted on medical students, with the majority of women in the US study (59%) with accurate knowledge that fertility peaked between the ages of 20 and 24 years, and 51% correctly identifying the period of significant fertility decline between the ages of 35 and 39 years [25]. About 75% of participants in a European study precisely indicated the optimal age for childbearing and the age at which fertility declined significantly [26].

#### Fertility risk factors

Five studies reported students’ awareness of fertility risk factors. These studies showed that college students had some understanding of fertility risk factors and could answer most of the risk factors correctly [6, 10, 16, 21, 26]. Most people are highly aware of the dangers of smoking and alcohol as bad habits for fertility [6, 10, 16, 21, 26]. At the same time, other lifestyle options (overeating, prolonged physical exertion, and irregular sleep patterns) were significantly less chosen. There is substantially less awareness of child-damaging behaviors, such as an unbalanced diet, excessive physical effort, and irregular sleep [21]. Two studies mentioned that students ranked age as a significant factor in fertility [6, 26]. A study in Argentina showed that students also recognized drug use (79.2%) and sexually transmitted infections (43%) as seriously affecting fertility [6]. Two studies identified the genetic disease factor [16, 21]. In summary, we observed that the level of awareness of students regarding fertility risk factors varies from medium to high.

#### Infertility definition and intended behavior in the event of infertility

Four studies assessed University students’ knowledge of the definition and related understanding of infertility. They found that they generally had limited knowledge of infertility problems and treatment and little sense of the intention of infertility and the associated causes of the disease [12, 15]. Many students (45.6%) answered that 40–60% of couples might experience difficulties conceiving, while 33.8% said it could be a problem for 20–40% [6]. 55% of women and 42% of men are aware of the prevalence of infertility [7].

A total of 12 studies investigated the intended behavior in the event of infertility. The study participants in the USA and European countries remained open to ARTs and were willing to undergo in vitro fertilization (IVF); no significant differences were observed between the sexes [7, 10–12, 20, 22, 23, 26]. Participants also reported the global trends in child adoption, with young people in Nigeria and Mexico and women in South Korea believing adoption is better than pursuing IVF [18, 24, 26]. In the case of infertility, the proportion of childless lifestyle choices is lower, and studies have shown that both men and women are less comfortable living childless [7, 10, 12, 23]. Surprisingly, younger people in Hong Kong are more likely to choose to remain childless and less likely to seek medical treatment or adoption than sample populations from the USA and Europe [17].

#### Knowledge about IVF treatments

Ten studies assessed University students’ knowledge of ARTs [6, 7, 9, 10, 17–19, 22–24]. The study showed that young people have a specific understanding of ARTs, such as IVF, with 26.8% of students saying they know what ART is, 39.2% saying that the probability of success of a single IVF cycle is < 40% and the majority (90%) said they understood the meaning of egg freezing [6].

However, young people were unable to assess the success rate of ART and showed undesirable confidence in the ability of fertility treatment to deliver positive results. A study in Saudi Arabia showed that only 22% of students overestimated the success rate of ART treatment [19], and 25% of young people in a Spanish study correctly answered the possibility of having a child through IVF [23]. Other studies were unsatisfactory, with 50% [23] to 75% [24] of participants overestimating the success rate of IVF and IVF with different techniques. In some countries with strong religious beliefs, ART is relatively unpopular.

### Influencing factors of FA

#### Gender

Six studies have shown that female students are more aware of fertility than male students, age-related fertility decline, and factors affecting fertility [7, 9, 10, 14, 21, 26]. Among these, a Swedish study showed that although women perceive fertility problems accurately, they are also likely to overestimate the chances of getting pregnant at ovulation and receiving a child through IVF [7]. Another four studies concluded that students generally lacked awareness of fertility issues, had low fertility awareness, and had no apparent differences between the sexes [11, 20, 23, 24].

#### Major

Five studies explored the differences in levels of FA among students in different majors [10, 12, 19, 21, 26]. Three of these studies compared the awareness of fertility issues between medical and non-medical students. They showed that medical students had higher levels of FA than students from other disciplines [10, 21, 26]. However, a study in Saudi Arabia showed that medical students were less aware of fertility, as only 52 (33.1%) of medical students believed that women had the easiest time having children at the age of 15–25 compared to 68.6% (P = 0.001) in other health professions [19]. Another study showed that science students are more aware of fertility than humanities students and have a more realistic view of fertility issues [12].

#### Education

Since this systematic review was on FA among college students, there was little difference in the educational level of the participants. Two studies compared the differences in the ranks of FA between undergraduate and graduate students, suggesting that years of schooling affect FA; people with high education had high levels of FA [21, 25].

#### Age

Only one study discussed the effect of age on FA, and the results showed that older participants had higher FA and a deeper understanding of fertility issues than younger participants [14].

## Discussion

Our review is a systematic evaluation using a summary of studies from the past decade to provide evidence on the overall level of FA among college students worldwide and the influencing factors. Although the studies we included were spread worldwide, a striking consistency was detected in the findings across the studies. This systematic evaluation suggested that University students globally had low FA and lacked appropriate knowledge of fertility issues. Although most people have heard of infertility, the specific definition and expertise are unclear. University students had some knowledge about fertility risk factors and could answer most of them correctly. They also considered age a significant factor affecting fertility; however, they overestimated the optimal period of human fertility and the age at which fertility begins to decline. Regarding ARTs, college students had some knowledge but were unable to assess the success rate of ARTs.

### Level of fertility awareness among college students

Our systematic evaluation found that students had little understanding of the concept of infertility, suggesting that students were unaware of some of the causes of the disorder. Although infertility has long been a public health issue of widespread concern, the link between college students and infertility has been understudied [27]. Many studies on college student fertility have focused on preventing unintended pregnancies, and most campus reproductive health services aim to prevent college student pregnancies [28]. It needs the attention of society and researchers because students in higher education are often nearing the age of declining fertility.

Young students can correctly identify some risk factors that affect fertility. Still, surprisingly they do not seem to be aware of how these lifestyle-related factors affect their fertility. A review by Pedro et al. found that people have a good understanding of lifestyle-related infertility risk factors (smoking, alcohol, and drug abuse) and a clear understanding that these factors are common risk factors for various other chronic diseases, such as cardiovascular disease and lung cancer [29]. College students learned about these risk factors more from health education about other conditions than from a fundamental understanding of their impact on fertility. Studies have shown that couples of childbearing age are increasingly aware that diet and nutrition may be related to reproductive performance in both men and women [30]. However, in younger student populations, students are less aware of the effects of diet and nutrition compared to poor lifestyle habits such as smoking and alcohol abuse. Studies from in vitro fertilization cohorts have further confirmed that healthy preconception dietary patterns may positively impact fertility [31]. Adherence to a healthy diet favoring fish, poultry, whole grains, fruit, and vegetables is associated with better fertility in women and better semen quality in men [32]. The relationship between vitamin D and fertility has been of interest to researchers. Although vitamin D deficiency may be detrimental to fertility, it is unclear whether higher vitamin D levels provide additional benefits once adequate levels are achieved [33, 34]. When young people leave the family environment and are removed from parental control, unhealthy eating habits such as reduced intake of fruit and vegetables, skipping breakfast, and increased intake of unhealthy snacks and fried foods can increase [35]. Greater precision in the relationship between diet, nutrition, and fertility is an increasingly important topic in reproductive health. Understanding the link between diet and fertility is essential for developing programs and behavior-change strategies to improve students’ lifestyles [36].

The results presented in this review raise concerns that college students may be delaying childbearing for social reasons but also for lack of awareness of age-related declines in human fertility. The students could recognize that age is a significant risk factor for fertility, yet their understanding of optimal fertility practices and the age at which fertility begins to decline for both men and women was low. Delaying childbearing is now a global trend, with a large population of young people waiting for their first birth until the age at which fertility begins to decline in pursuit of higher education and better jobs [37]. Involuntary childlessness and secondary infertility can become severe problems if students make family planning decisions based on their basic knowledge of fertility issues.

Along with technological advances, young people believe in modern medical technology, and most male and female students are willing to undergo ART under the assumption of infertility. However, they might overestimate the success rate of medical technology [38]. This finding was consistent with our pooled evidence that young students overestimate the success rate of ART. In addition to the influence of age factors, accidental complete fertilization failure is still an unfortunate event, and fertilization failure may be caused by oocytes or sperm. Implicit sperm defects in immature oocytes and significantly normal sperm may be the cause of fertilization failure [39]. Untreated chronic anovulation is one of the main causes of female infertility, which can induce estrogen deficiency [40]. Oral ovulation inducers are the first-line treatment for most anovulatory patients. Research has shown that inositol (MI) and d-inositol (DCI) play crucial roles in ovarian physiology [41]. Research on patients undergoing assisted reproductive surgery has found that both compounds are essential [42, 43] and can achieve optimal oocyte development through specific ratios [44, 45]. Clinical cases have reported 2 cases of young oligomenorrheic women with such characteristics who ovulated after receiving a high-dose (1200 mg) of d-chiro-inositol treatment for 6 weeks [40]. Thyroid autoimmunity (TAI) is also considered one of the main causes of female infertility related to reduced ovarian reserve and potential damage to oocyte maturation and embryonic development, leading to adverse pregnancy outcomes. More and more evidence emphasizes its impact on natural pregnancy and in vitro fertilization [46].

The implantation of human embryos in the in vitro fertilization/ICSI cycle is a black hole in current knowledge, among other factors, embryo transfer (ET) technology is considered an important determinant of success rate. According to reports, pre-endometrial injection of embryo culture supernatant can improve implantation and pregnancy rates [47]. Studies have shown that injecting embryo culture supernatant into the uterine cavity 30 days or 5 days before embryo transfer not only improves the pregnancy rate of IVF/ICSI or OD cycles but also has no adverse effects [48].

### Influencing factors of FA

Research has found that gender, major, education level, and age are the main influencing factors. Six articles indicate that female students have higher overall A levels than male students. Unsurprisingly, household women often participate more in pre-pregnancy care and pay attention to fertility. A Swedish study showed that women value parenthood (naturally, through adoption or IVF) more than men [49]. Although women and men have similar personal intentions for childbirth, women believe that having a child is much more critical than men. In the case of infertility, women are also more likely to undergo IVF treatment or adoption [7]. Gender models influence women’s and men’s general and reproductive behavior [50, 51]. Although both women and men are troubled by childbirth, women’s psychological reactions are influenced by their gender [52, 53]. Therefore, women will pay more attention to childbirth-related information and are more likely to receive fertility-related knowledge. A 2002 study of 1385 adolescent boys in Tehran showed a poor understanding of sexual and reproductive health (SRH) [54]. Four other articles suggest that no significant differences were found between male and female students, suggesting that the association between gender and FA requires more research to validate. The study indicates that both boys and girls overlook the seriousness of involuntary infertility and that young people tend to be more interested in completing their studies and choosing jobs, leading modern young people to delay childbearing [55]. Students significantly overestimated female fertility and were unaware of age-related decline in fertility. Women may value fertility more than men, but their perception of age-related fertility is less accurate. They are more likely to overestimate the chances of pregnancy at ovulation and the option of receiving a child through IVF. In addition, women’s response rates were higher overall than men’s. The proportion of female participants was significantly higher than that of men in most study samples, which may have contributed to significant gender factors.

This systematic review found a correlation between students’ majors and their level of awareness. As expected, the story of FA was significantly higher among medical students than non-medical students. Although medical students are prone to overestimate their fertility potential because fertility education is present in medical specialty education programs, medical students are more knowledgeable and realistic about fertility issues, and they have a better understanding of risk factors for fertility and positive attitudes toward ARTs. In addition, despite being more aware of fertility issues than non-medical students, medical students still tend to delay family planning and want to have fewer children because of solid conflicts between medical careers and their fertility and family plans. These findings suggested that non-medical students need specialized fertility education, while medical students need support to secure their medical jobs and family needs.

Students reported learning about reproductive health primarily from social media and acquiring some knowledge. Still, the poor coverage and depth of expertise indicated that they could not make informed decisions about their fertility [56]. A few people reported that they learned about it from a medical professional; hence, there are questions about which reproductive health services are available to doctors and how much the students learned from them. The importance of fertility information cannot be overstated; the data should be significantly improved to enable young people to make informed decisions about life planning.

### How to improve fertility awareness

During compulsory education, relevant information should be provided to students who have not yet entered University. To emphasize the importance of fertility knowledge, general practitioners, obstetrician-gynecologists, and nurses should conduct popular science education before young students start sex. In addition, schools should also assume corresponding mission responsibilities and incorporate reproductive education into their daily instruction. The media should exercise caution in providing specific information about human reproduction and exclude incorrect and inappropriate content.

Of course, we should not limit ourselves to traditional educational programs. Because with the development of the times, traditional methods of reproductive education may not be able to meet the needs of society and people of childbearing age. At present, artificial intelligence (AI) has gradually been applied in many fields of medicine. By combining knowledge with computer science through machine learning algorithms, AI has the potential to improve infertility diagnosis and ART estimation results [57]. Bachelot et al. [58] conducted a study in which they proposed a highly promising machine learning model that can stratify infertile/fertile couples based on their biological clinical characteristics, thereby helping to manage unexplained infertile couples. Mobile health applications such as “Smart Pregnancy” [59] and virtual animated characters [60] have shown promising results in improving pre-pregnancy health. Japanese scholars using educational chatbots to understand fertility and pre-pregnancy health have significantly increased women’s fertility knowledge and changed their intentions, optimizing pre-pregnancy health immediately after contact [61]. These fully demonstrate that new digital technologies can provide more choices for fertility and pre-pregnancy health education. In future research on fertility and reproductive health, we should continue to conduct further technological development and research, exploring individuals’ affinity for technology in fertility awareness. It is worth noting that human autonomy in the context of healthcare must always be given priority and cannot break through the bottom line of medical ethics.

## Conclusions

In recent years, several researchers have begun to study fertility awareness, but the quality of the results is uneven and lacks heterogeneity. This systematic review found low to moderate levels of fertility awareness among University students but higher levels among women and medical students. In addition, the results of most studies are a significant concern as college students may delay childbearing, which is related to social reasons but also a lack of awareness of the age-related decline in human fertility. Government and educational institutions should strengthen the education programs for young students on reproductive health to raise awareness about childbirth, and society should provide family support to young people.

## Limitations

This systematic review conducted a study search using four databases to obtain the best-published evidence on college students’ FA. In addition, two independent researchers conducted study selection and quality assessment, and we critically assessed the findings. Next, we included studies from four continents and interpreted the results globally.

Nevertheless, the present study has some limitations. First, the different sample sizes of each study led to heterogeneity. Second, some of the studies used other and uneven fertility awareness assessment instruments, which led to questionable credibility of the results from each study. The most commonly used fertility awareness scale, developed by Swedish investigators, contains multiple dimensions, and the entries are primarily open-ended questions that lack direct assessment. Third, most existing studies are from Europe, with only a few reflections from other regions, which produces ethnic and cultural differences that increase the potential for bias.

In future research, several issues need to be addressed: (1) developing a valid and reliable fertility awareness assessment tool; (2) conducting fertility awareness studies in multiple regions to understand the perception of college students about fertility worldwide; (3) focusing on the factors influencing fertility awareness and determining how to develop appropriate fertility awareness programs through specific linkages.

## Supplementary Information

**Additional file 1.** PRISMA 2020 checklist.

## Data Availability

All data generated or analyzed during this study are included in this published article.
