# Supplementary material for: University students’ fertility awareness and its influencing factors: a systematic review
Source: Reprod Health. 2023 Jun 6;20:85. doi: 10.1186/s12978-023-01628-6 (PMC10242772; doi:10.1186/s12978-023-01628-6)
Supplement: Supplementary file 1 — Additional file 1. PRISMA 2020 checklist. [file 12978_2023_1628_MOESM1_ESM.docx]

| **Section and Topic** | **Item #** | **Checklist item** | **Location where item is reported** |
| --- | --- | --- | --- |
| **TITLE** | | |  |
| Title | 1 | University students' fertility awareness and its influencing factors: a systematic review | Page 1 |
| **ABSTRACT** | | |  |
| Abstract | 2 | Introduction: In recent years, a growing number of researchers have begun to study fertility awareness (FA). Evidence suggests that college students in their reproductive years have low awareness of fertility, risk factors for infertility, and assisted reproductive technologies. Therefore, this systematic review attempts to summarize these studies and explore the influencing factors that affect college students' fertility awareness.Methods:A systematic literature search of databases (PUBMED/MEDLINE, Cochrane, Web of science, Embase and EBSCO) were conducted from inception to September 2022. Studies that assessed the levels of fertility awareness and factors influencing college students were considered for the review. The qualities of the included studies were evaluated using the Strengthening the Reporting of Observational Studies in Epidemiology guidelines. This systematic review is reported according to the preferred reporting items for systematic review (PRISMA) guideline. Results:Twenty-one articles met the eligibility criteria and were included. The primary results showed that participants reported low to moderate FA. female, medical students demonstrated higher levels of fertility awareness. The association between age, years of education, and FA was not sufficient.Conclusion: The results of the current study suggest that increased FA interventions are warranted, especially for the male, non-medical student population.Governments and educational institutions should strengthen education programs for young students on reproductive health to help them raise awareness about childbirth, and society should provide family support for young people. | Page 1-2 |
| **INTRODUCTION** | | |  |
| Rationale | 3 | In recent years, several studies have investigated fertility awareness (FA). Accumulating evidence suggests that college students in their reproductive years have low awareness of fertility, risk factors for infertility, and assisted reproductive technologies (ARTs). Therefore, this systematic review attempts to summarize these studies and explore the influencing factors that affect college students' FA. | Page 2-3 |
| Objectives | 4 | This systematic review attempts to summarize these studies and explore the influencing factors that affect college students' fertility awareness. | Page 3 |
| **METHODS** | | |  |
| Eligibility criteria | 5 | The eligible studies had to meet all of the following criteria: 1) Research subjects were University students; 2) Quantitative data on FA (for example, age-related fertility decline, fertility risk factors, infertility definition, and intended behavior in the event of infertility, knowledge about IVF treatments, and influencing factors of fertility awareness) were used; 3) FA-specific measures were used or the problem of the evaluation was described in detail.We excluded studies of the following criteria:Non-English publications, reviews, abstracts, communications, case reports, and studies that could not be provided in full and non-human studies were excluded. | Page 4 |
| Information sources | 6 | We performed searches in the following databases:  o Embase (Excerpta Medica dataBASE)  o Cochrane Library  o Web of Science  o PubMed  o EBSCO  In addition, relevant citations from the included studies were searched by hand. | Page 3-4 |
| Search strategy | 7 | Present the full search strategies for all databases, registers and websites, including any filters and limits used. | Page 3 |
| Selection process | 8 | In this study, two researchers completed the screening of information in the literature. After excluding the inconsistent literature, they continued reading the rest of the literature to determine if it could be included. The above tasks were done by two people independently. Finally, two researchers cross-checked the results, and if there was any disagreement during the period, a third party could be involved to discuss and resolve the disagreement. Missing or uncertain literature could be obtained by contacting the author by email. | Page 4 |
| Data collection process | 9 | All searched articles were first imported into NoteExpress software to automatically remove the duplicates, and then two reviewers screened the titles and abstracts of the studies according to inclusion and exclusion criteria to exclude irrelevant articles. Articles that met the preliminary eligibility criteria were subjected to full-text screening by the same two reviewers. Any disagreements were resolved by consulting with a third reviewer. | Page 4 |
| Data items | 10a | We presented the major outcomes below in the ‘Summary of findings’ tables.  • University students globally have low FA .  • University students lack proper knowledge of fertility issues.  • University students overestimate the optimal age of human fertility and the age at which fertility begins to decline and were unable to assess the success rate of ARTs.  • Females and medical students demonstrated higher levels of FA, but the association between age, years of education, and FA was not sufficient.  We extracted outcome indicators that assess influencing factors of FA:  • gender;• major; • education; • age. | Page 4 |
|  | 10b | The data were extracted using an Excel sheet designed in advance by two reviewers , and any disagreements were resolved by a third reviewer. The following parameters were recorded: 1) author and year of publication, 2) country, 3) study type, population, and sample size, 4) measurement tools used, 5) mean age, 6) education level, 7) influencing factors, and 8) primary findings. | Page 4 |
| Study risk of bias assessment | 11 | The quality of each eligible article was independently assessed by two reviewers using the Guidelines for Strengthening Epidemiological Observational Studies (University of Bern, 2009). The guidelines consisted of 22 items to assess the quality of cross-sectional and case-control articles. Each item was scored 1 if the study met the criteria of the guidelines and 0 if the study described the item inadequately; the overall maximum score was 22 points. Studies with an overall score of ≥17 were considered high quality, those with an overall score between 11 and 16 were rated as moderate quality, and those with a total score of 10 were considered low quality. A quality assessment of 21 included quantitative studies did not identify any low-quality studies. Of these, 20 were considered high quality, while one study was rated as moderate quality. The distribution of scores is listed in Table 2. | Page 5 |
| Effect measures | 12 | Based on the information obtained from the included studies, we assessed the levels of fertility awareness and factors influencing university students through a tabular summary. The results section of the table describes the key conclusions of each included study. | Page 5 |
| Synthesis methods | 13a | The studies included in this systematic review are quantitative , and the quantitative studies are cross-sectional observational studies, and the important results of each study are integrated using tabular descriptions. | Page 5 |
|  | 13b | By combining information from different research backgrounds, all aspects of the research problem are explored, and the contributions of each original research are described in turn, and the contradictory parts are explained. |  |
|  | 13c | We compiled a "Summary of Results" table using Excel documents. These tables include the source of the study, the number of participants, and a summary of the study for each major outcome. We summarize the levels of fertility awareness and factors influencing university students. |  |
|  | 13d | This review summarized the key findings in the article using a tabular summary. |  |
|  | 13e | No sensitivity analysis was performed. |  |
|  | 13f | No sensitivity analysis was performed.. |  |
| Reporting bias assessment | 14 | To assess selective reporting bias, we compared the results hypothesized by the original investigator during the study period with the reports in the published papers by comparing the information section in the final publication. If there are no published study protocols, and the authors do not provide unpublished protocols as requested, we compared the methodological and results sections of published papers. We also use our knowledge of the clinical field to identify places where researchers are not reporting commonly used outcome measurements | Page 5 |
| Certainty assessment | 15 | This study is not applicable. | N |
| **RESULTS** | | |  |
| Study selection | 16a | Fig. 1 summarizes the study selection process. A total of 704 studies were retrieved in the initial search, and 616 studies were retained after removing the duplicates. Subsequently, 504 studies were deemed irrelevant and excluded after screening the titles and abstracts. | Page 5 |
|  | 16b | We carefully reviewed the full text of 112 studies, of which 91 were excluded for a variety of reasons. Finally, 21 studies were included in the systematic review |  |
| Study characteristics | 17 | The review included 21 studies, with 18 cross-sectional studies and 3 case-control articles(table 1). | Page 6 |
| Risk of bias in studies | 18 | The studies included in this review were cross-sectional and case-control articles, and no risk assessment was conducted. | N |
| Results of individual studies | 19 | The results of the levels of fertility awareness and factors influencing university students were summarized using the table (table 1). | Page 6-10 |
| Results of syntheses | 20a | None | Page 6-10 |
|  | 20b | This systematic review uses tables to summarize the results of the levels of fertility awareness and factors influencing university students. |  |
|  | 20c | The studies included in this review were cross-sectional and case-control articles, with no heterogeneous manifestations. |  |
|  | 20d | The studies included in this systematic review were cross-sectional and case-control articles, and no sensitivity analyses were conducted. |  |
| Reporting biases | 21 | The studies included in this systematic review were cross-sectional and case-control articles and did not report bias. | N |
| Certainty of evidence | 22 | Two investigators assessed the certainty (or confidence) of the body of evidence for each outcome assessed. | N |
| **DISCUSSION** | | |  |
| Discussion | 23a | Provide a general interpretation of the results in the context of other evidence. | Page 10-15 |
|  | 23b | Discuss any limitations of the evidence included in the review. |  |
|  | 23c | Discuss any limitations of the review processes used. |  |
|  | 23d | Discuss implications of the results for practice, policy, and future research. |  |
| **OTHER INFORMATION** | | |  |
| Registration and protocol | 24a | The current protocol is registered with the Open Science Framework | Page 3 |
|  | 24b | The review protocol was registered with the International Prospective Register of Systematic Reviews (PROSPERO) database (registration number: CRD42022372075) and the protocol has been published . |  |
|  | 24c | No revisions were made to the information provided at the time of registration or in the protocol. |  |
| Support | 25 | 1.National Natural Science Foundation of China (Grant number 82101674)  2.Natural Science Foundation of Jiangsu Province, China (Grant number BK20210815). | Page 17 |
| Competing interests | 26 | None | N |
| Availability of data, code and other materials | 27 | The study data can be requested from the author. The articles for this review can be made available upon request. | Page 17 |

*From:*  Page MJ, McKenzie JE, Bossuyt PM, Boutron I, Hoffmann TC, Mulrow CD, et al. The PRISMA 2020 statement: an updated guideline for reporting systematic reviews. BMJ 2021;372:n71. doi: 10.1136/bmj.n71

For more information, visit: <http://www.prisma-statement.org/>
